# Determinants of infant and young complementary feeding practices among children 6–23 months of age in urban Pakistan: a multicenter longitudinal study

**DOI:** 10.1186/s40795-020-00401-3

**Published:** 2020-12-16

**Authors:** Shabina Ariff, Kamran Saddiq, Javairia Khalid, Laila Sikanderali, Batha Tariq, Fariha Shaheen, Gul Nawaz, Atif Habib, Sajid Bashir Soofi

**Affiliations:** Department of Paediatrics and Child Health, Aga Khan University, Karachi, Pakistan

## Abstract

**Background:**

Suboptimal feeding practices have a negative impact on children’s health and growth in the first 2 years of life and increase their risk of undernutrition, morbidity, and mortality. The aim of the study was to assess the factors that influence infant and young child feeding practices among urban mothers in a hospital setting at Karachi, Pakistan.

**Methods:**

A longitudinal multi-center cohort study was conducted in four countries, MULTICENTER BODY COMPOSITION REFERENCE STUDY (MBCRS) to produce normal body composition reference data in healthy infants from 3 months to 24 months of age. Repeated anthropometric (weight, length and head circumference) and body composition measurements using “deuterium dilution method” along with 24-h dietary recall questionnaires were performed on 250 healthy term infants at 3, 6, 9, 12, 18, and 24 months of age. The 24-h dietary recall data from this study was used to assess the breastfeeding and complementary feeding practices in children aged 6–24 months.

**Results:**

A total of 250 healthy infants were enrolled in the study. A majority of newborns (75.4%) were exclusively breastfed till 3 months of age; however, by 6 months of age, only 30.2% of infants were exclusively breastfed. Only 44.1% of children aged 6–24 months achieved minimum dietary diversity (MDD), 84.7% achieved minimum meal frequency (MMF), and 44.1% achieved a minimum acceptable diet (MAD). 71.4% achieved MDD and MAD and 100% achieved MMF at 24 months. The bivariate analysis found that breastfed children (OR 3.93, 95% CI 2.72–5.68), with employed mothers (OR 1.55, 95% CI 1.06–2.27) who had graduated from secondary school (OR 1.45, 95% CI 1.08–1.94) were more likely to meet minimum dietary diversity. The multivariable analysis showed that only the child’s age was significantly associated with MDD (*p* value< 0.0001), with the likelihood of meeting MDD increasing as the children aged; 9 months (OR 18.96, 95% CI 6.63–54.19), 12 months (OR 40.25, 95% CI 14.14–114.58), 18 months (OR 90.02, 95% CI 30.84–262.77) and 24 months (OR 82.14, 95% CI 27.23–247.83).

**Conclusion:**

Our study revealed that Infant and young child feeding practices are significantly associated with maternal education, employment, and the child’s age. Therefore, it is essential that investments be made towards protective breastfeeding and complementary feeding policies and legislations, emphasis on female education and ensuring the availability of affordable nutritious and diverse foods.

## Strength and limitations

We were able to study determinants of IYCF practices in an urban population.

Majority of our respondent mothers willingly participated in the study.

We used 7 food groups, recommended by the WHO to measure dietary diversity.

Data on feeding practices was collected on the 24-h recall only and consumption precision may not have been absolute.

## Introduction

Recent global estimates have shown a slight decline in the prevalence of child stunting (21.3%) and wasting (6.9%); however, undernutrition remains an alarming concern as the decline has been slow and continues to impact the lives of millions of children under 5 years [1] . According to regional estimates, South Asia was revealed to have one of the highest burdens of undernutrition (31.7% stunted; 14.3% wasted) [1]. While among South Asian countries, Pakistan is ranked as having the highest prevalence of stunting (40.2%), wasting (17.7%) and underweight (28.9%) [2–8]. Inadequate breastfeeding and suboptimal complementary feeding practices are consistent predictors of malnutrition in the first 2 years of life [9]. This period has been recognized as the ‘critical window of opportunity’ for the promotion of optimal growth, health and development of a child [10]. Children may become malnourished if they are not exclusively breastfed during the first 6 months of life, and do not receive sufficient quantities of nutritionally adequate complementary foods after 6 months of age with continued breastfeeding till 2 years of age [11].

In Pakistan, exclusive breastfeeding (48.4%), complementary feeding practices, such minimum dietary diversity (MDD) (14.2%), minimum meal frequency (MMF) (18.2%) and minimum acceptable diet (MAD) (3.6%), and continued breastfeeding till 2 years (56.5%) are far below acceptable levels [7]. Lack of dietary diversity among children is a significant problem across the country, where diets predominantly consist of starchy staples with limited flesh foods, seasonal fruits and vegetables [12, 13]. Inadequate breastfeeding and complementary feeding practices, also referred to as infant and young child feeding (IYCF) practices, leave children vulnerable to malnutrition in the first 2 years of life [14]. “Majority of the studies” conducted in Pakistan primarily focus on identifying determinants of IYCF practices in rural communities, where community health workers provide monthly social and behavioural change communication messages related to maternal nutrition, IYCF practices, health and hygiene. To address the gap in evidence among urban communities, data from the Multicenter Body Composition Reference Study (MBCRS) was used to conduct a secondary analysis of IYCF practices among children 6–23 months [15]. The current study assessed the factors that influence IYCF practices among urban mothers in a hospital setting at the Aga Khan University Hospital, Karachi, Sindh, Pakistan.

## Methodology

### Study design and setting

This was a nested cross-sectional study within the large parent study of MBCRS, carried out at the Aga Khan University Hospital a tertiary care center with multiple outreach hospitals, providing a wide range of clinical services across Pakistan. The study was carried out in collaboration with the International Atomic Energy Agency (IAEA) from October 2014 to November 2017. The objective of the MBCRSstudy was to produce normal body composition reference data in healthy infants from 3 months to 24 months of age. Repeated anthropometric (weight, length and head circumference) and body composition (deuterium dilution method) measurements along with 24-h dietary recall questionnaires were performed on 250 healthy term infants at 3, 6, 9, 12, 18, and 24 months of age. The 24-h dietary recall data from this study was used to assess the breastfeeding and complementary feeding practices in children aged 6–24 months. The mothers were also provided with routine SBCC messages related to maternal nutrition, IYCF practices, health and hygiene practices during their monthly post-natal follow-up visits tothe hospital .

### Sample size

The Sample size was calculated on the basis of the primary outcome. The proposed study centres have a power of 90% to detect fat mass and far free mass for boys and girls less than one standard deviation away from a reference study [16]; United States; mean FM 3.10 ± 0.5 kg and 3.05 ± 0.46 kg for boys and girls respectively; and mean FFM 9.13 ± 1.06 kg and 8.99 ± 1.1 kg for boys and girls respectively). Sites (*n* = 4) and study population (*n* = minimum 400) within 2 years. Pakistan (*n* = 150), Brazil (*n* = 150), South Africa (*n* = 150), Australia (*n* = 150).

### Study participants and eligibility criteria

Pregnant mothers at least 18 years or above, who had given birth between 37 weeks (early term) to 41 weeks (late term) of gestation, intended to exclusively breastfeed for the first 6 months and preferably continue for at least 12 months, attained at least a secondary level education, and were non-smokers with no significant morbidity, were invited to participate in the study. The mother and infant dyads were enrolled from the labour and delivery ward between October 2014 – October 2015 and followed up till November 2017 for main study MBCRS. Written consent was obtained from mothers prior to enrolment in the study.

### Data collection

A structured questionnaire was administered to collect information from mothers on demographic characteristics, reproductive history, gestational age, infant’s dietary intake and anthropometry by trained interviewers. A multi-pass 24-h dietary recall was used to obtain in-depth data on the type and quantity of all beverages and foods consumed by the infant during the past 24 h [17]. The multi-pass approach has been validated in many countries and has been found to increase the interviewer’s ability to collect substantial details on each beverage or food, along with its preparation method and the portion sizes consumed [17]. Data collected from the 24-h recall was used to assess the Minimum dietary diversity (MDD), minimum acceptable diet (MAD) andMinimum meal frequency (MMF), while the repeated recalls led to the identification of a usual intake among infants living in urban areas.

The study data collection team consisted of a team supervisor and five data collectors. Due to local cultural restrictions, a female medical officer and female nurses were hired as the team supervisor and data collectors. The data collection team received 5 days of training on the study methods, questionnaires, anthropometric measurements, deuterium dilution techniques, and ethical issues. This was followed by 2 days training on data collection form for Food recall and food frequency and standardization training on anthropometry using the INTERGROWTH-21stInternational Fetal and Newborn Growth Standards for the twenty-first Century, The International Fetal and Newborn Growth Consortium, INTERGROWTH-21st Anthropometry Manual, University of Oxford. The study questionnaire was pre-tested, and changes incorporated prior to data collection. For quality assurance, the team supervisor and study manager randomly reassessed and validated the data collected from 5% of mother and infants. The Emergency Nutrition Assessment software was used to conduct automated plausibility and data quality checks for the anthropometric data.

### Statistical analysis

Descriptive statistics related to socioeconomic status, education, and health were presented as mean ± standard deviation (SD), frequencies and percentages. Furthermore, a chi square test was used to determine an association between variables. Logistic regression was used to assess the relationship between IYCF practices with other variables. World Health Organization (WHO) standard indicators for assessing IYCF practices were used to determine adequate meal frequency, diversity and acceptability [13]. To assess the minimum dietary diversity, dietary recall data was classified using the seven WHO food groups (Table 1). Odds ratios (OR) were reported with 95% confidence intervals (CI). Significance has been defined as *P* value < 0.05. Data were double entered in the Visual FoxPro database by trained data input operators. All analyses was performed in SPSS version 15.

**Table 1:** Classification of foods consumed by children according to WHO’s seven food groups

Food groups	Foods mentioned in infant 24-h recall form
Grains, roots and tubers	Rice, Tuber (potato), Bread/Cookies/Crackers, Porridge/Cereal, Pasta
Legumes and nuts	Beans
Dairy products	Milk, yoghurt, cheese
Flesh foods	Red/Organ meats (Beef, Liver, Kidney), Fish, Poultry
Eggs	Eggs
Vitamin A rich fruits and vegetables	Peaches, watermelon, mangoes, carrots, leafy vegetables, sweet potatoes,
Other fruits and vegetables	Various

## Results

### Characteristics of study population

A total of 250 pregnant mother and newborn dyads were enrolled in the study and followed up till the infant’s second birthday. About half (53.2%) of the enrolled mothers and infants completed the study in the main study. A total of 117 mothers were lost to follow up due to multiple reasons including migration, unavailable etc. Among the enrolled children, 118 (47.2%) were males and 132 (52.8%) were females (Table 2). The average birthweight was 3.11 (± 0.37) kg and the length and head circumference were 49.05 (± 1.67) cm and 33.91 (± 1.05) cm, respectively. The mean age of the mothers was 28.48 (±4.4) years. Majority of the mothers (80.4%) were housewives, with an average 14.39 ± 2.53 years of education. Approximately, 105 (42.0%) were first-time mothers, 81 (32.4%) were second-time mothers and 64 (25.6%) already had two or more children. A similar proportion of families was observed across the average monthly household’s income categories; 38.8% had an income between 50,000–75,000 PKR, 29.2% had an income between 75,000–100, 000 and 32.0% has an income > 100,000PKR.

**Table 2:** Characteristics of study infants and their mothers (*N* = 250)

Baseline characteristics	Frequency (%)
Infant Characteristics	
Gender	
Female	132 (52.8)
Male	118 (47.2)
Birth weight (kg)	3.11 ± 0.37
Birth length (cm)	49.05 ± 1.67
Birth head circumference (cm)	33.91 ± 1.05
Maternal Characteristics	
Mother’s age (years)	28.48 ± 4.4
Formal years of education	14.39 ± 2.53
Highest level of education	
≤ 14 years	126 (50.4)
15–16 years	90 (36)
> 16 years	34 (13.6)
Occupation	
Unemployed	201 (80.4)
Employed	49 (19.6)
Average monthly household income	
50,000–75,000 PKR	97 (38.8)
75,000–100,000 PKR	73 (29.2)
> 100,000 PKR	80 (32.0)
Number of previous births	
0	105 (42)
1	81 (32.4)
2	45 (18)
> 2	19 (7.6)

N.B. Data presented as mean ± SD and n (%), *PKR* Pakistani rupees

### Breastfeeding and complementary feeding practices

A majority of newborns (75.4%) were exclusively breastfed till 3 months of age; however, by 6 months of age, only 30.2% of infants were exclusively breastfed (Table 3). Solid, semi-solid and soft foods were introduced to infants, on average, by 5.44 ± 0.56 months, with 60.4% of infants being introduced to these foods at 6 months of age. Age appropriate breastfeeding was found to be 81.1% among children 0–23 months of age.

**Table 3:** Breastfeeding and complementary feeding practices pattern of the study infants

IYCF Indicators	No (%)
Exclusively breastfed at 3 months (*n* = 171)	129 (75.4)
Exclusively breastfed at 6 months (*n* = 159)	48 (30.2)
Predominantly breastfed at 6 months (*n* = 159)	15 (9.4)
Age appropriate breastfeeding (*n* = 159)	129 (81.1%)
Continued breastfeeding at 1 year of age (*n* = 154)	136 (88.3)
Continued breastfeeding at 2 years of age (*n* = 133)	43 (32.3)
Average age at introduction of solid, semi-solid or soft foods	5.44 ± 0.56
Introduction of solid, semi-solid or soft foods at 6 months of age (*n* = 159)	90 (60.4%)

**Fig. 1:**
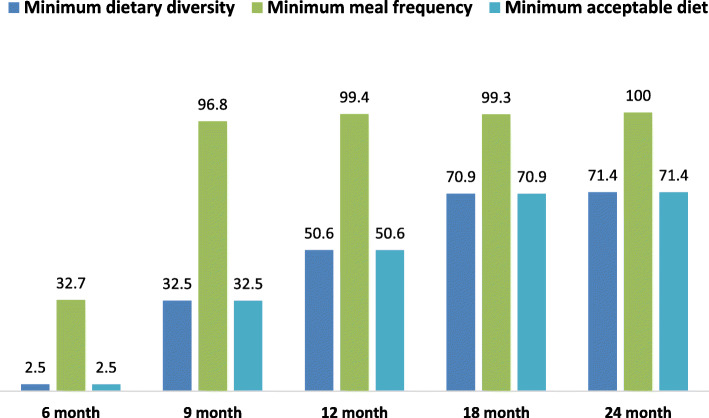
Overall percentage of children with minimum dietary diversity, meal frequency and acceptable diets calculated at 6, 9, 12, 18, and 24 months of age

A total of 88.3% of infants continued to be breastfed at 12 months of age, while at 2 years of age 32.2% of infants continued to be breastfed. Only 44.1% of children aged 6–23 months achieved minimum dietary diversity (MDD), 84.7% achieved minimum meal frequency (MMF), and 44.1% achieved a minimum acceptable diet (MAD). Children were found to increasingly achieve the three complementary feeding indicators as they aged, with 71.4% achieving MDD and MAD and 100% achieving MMF at 24 months of age (Fig. 1).

**Fig. 2:**
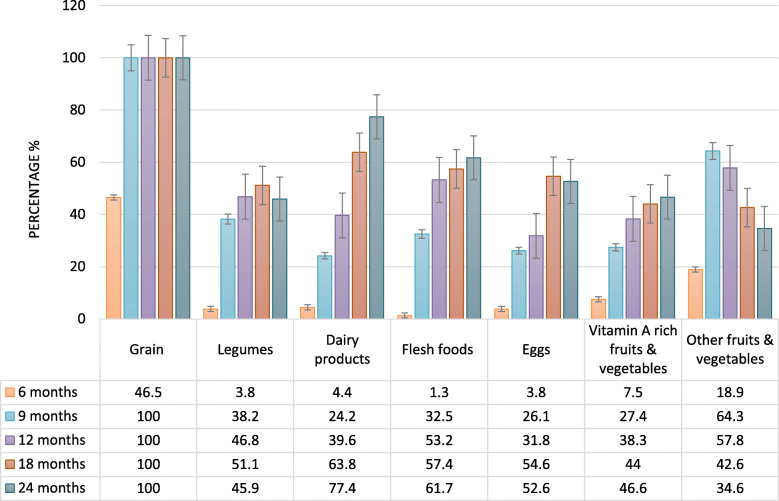
Frequency of consumption of various food groups in %, according to WHO recommendations, across different age groups of study participants

Children 6–23 months were found to have consumed foods on average from 2.56 (± 1.99) food groups of the seven WHO food groups. At 6 months of age, children primarily consumed grains, roots and tubers (46.5%) and other fruits and vegetables (18.9%), followed by vitamin A rich fruits and vegetables (7.5%), dairy products (4.4%), eggs (3.8%), legumes and nuts (3.8%) and flesh foods (1.3%) (Fig. 2). From 9 to 12 months of age, children were found to mainly consume grains, roots and tubers (100%) and other fruits and vegetables (~ 60%), with a substantial increase in consumption of flesh foods and legumes and nuts. However, from 18 to 24 months of age, these children consumed predominately grains, roots and tubers, flesh foods and dairy products.

**Fig. 3:**
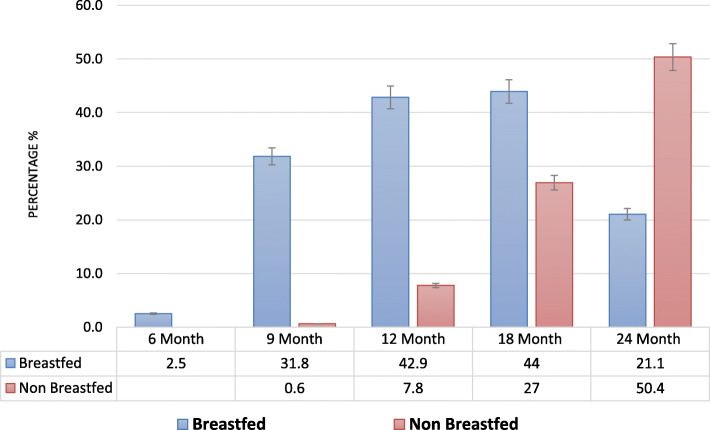
Patterns in dietary diversity among breastfed and non-breastfed children, assessed at 6, 9, 12, 18 and 24 months of age

The consumption trends from these food groups indicate that as the children aged, they were provided with a more diverse diet. The consumption trends were similar among the food groups, with the exception of other fruits and vegetables. Once stratified by breastfeeding practices (breastfed; non-breastfed), older children were found to consume a more diverse diet as compared to younger children (Fig. 3).

### Factors associated with minimum dietary diversity among children aged 6–23 months

The odds ratio (OR) and 95% CI for factors associated with MDD in the univariate and multivariable models are presented in Table 4. The univariate analysis showed that breastfed children (OR 3.93, 95% CI 2.72–5.68), with employed mothers (OR 1.55, 95% CI 1.06–2.27) who had graduated from secondary school (OR 1.45, 95% CI 1.08–1.94) were more likely to meet minimum dietary diversity. Children at 9 months (OR 18.64, 95% CI 6.52–53.13), 12 months (OR 39.77, 95% CI 14.03–112.69), 18 months (OR 94.51, 95% CI 32.84–271.98) and 24 months (OR 96.87, 95% CI 33.51–280.02) when compared to children at 6 months (reference group) were more likely to meet MDD. There was no association between the child’s gender, maternal age, birth order, household income and the likelihood of a child meeting MDD. The multivariable analysis examined the likelihood of breastfeeding practices, child’s age, maternal education, employment, and household income influencing the child’s ability to meet MDD. The multivariable analysis shows that only the child’s age was significantly associated with MDD (*p* value< 0.0001), with the likelihood of meeting MDD increasing as the children aged; 9 months (OR 18.96, 95% CI 6.63–54.19), 12 months (OR 40.25, 95% CI 14.14–114.58), 18 months (OR 90.02, 95% CI 30.84–262.77) and 24 months (OR 82.14, 95% CI 27.23–247.83).

**Table 4:** Factors associated with minimum dietary diversity among children aged 6–23 months

	Dietary diversity			Univariate analysis		Multivariable analysis	
	No	Yes	Total	OR (95% CI)	***P***-value	OR (95% CI)	***P***-value
**Breastfeeding**							
Yes	52 (30.6)	118 (69.4)	170	3.93 (2.72–5.68)	< 0.0001	1.4 (0.88–2.22)	0.158
No	364 (63.4)	210 (36.6)	574	Ref		Ref	
**Age**							
6 months	155 (97.5)	4 (2.5)	159	Ref.		Ref	
9 months	106 (67.5)	51 (32.5)	157	18.64 (6.52–53.13)	< 0.0001	18.96 (6.63–54.19)	< 0.0001
12 months	76 (49.4)	78 (50.6)	154	39.77 (14.03–112.69)	< 0.0001	40.25 (14.14–114.58)	< 0.0001
18 months	41 (29.1)	100 (70.9)	141	94.51 (32.84–271.98)	< 0.0001	90.02 (30.84–262.77)	< 0.0001
24 months	38 (28.6)	95 (71.4)	133	96.87 (33.51–280.02)	< 0.0001	82.14 (27.23–247.83)	< 0.0001
**Gender**							
Female	211 (54.7)	175 (45.3)	386	1.11 (0.83–1.48)	0.476		
Male	205 (57.3)	153 (42.7)	358	Ref.			
**Maternal education**							
≤ 14 Years	225 (60.5)	147 (39.5)	372	Ref.		Ref.	
> 14 Years	191 (51.3)	181 (48.7)	372	1.45 (1.08–1.94)	0.012	1.41 (0.96–2.07)	0.078
**Mother’s age**							
15–18 years	3 (60)	2 (40)	5	Ref.			
19–34 years	373 (56.2)	290 (43.7)	663	1.16 (0.19–7.02)	0.867		
≥ 35 years	40 (52.6)	36 (47.4)	76	1.35 (0.21–8.54)	0.750		
**Number of children**							
First child	164 (56)	129 (44)	293	Ref.			
< 2	135 (53.8)	116 (46.2)	251	1.09 (0.78–1.53)	0.609		
≥ 2	117 (58.5)	83 (41.5)	200	0.9 (0.63–1.30)	0.578		
**Mother’s occupation**							
Unemployed	356 (57.8)	260 (42.2)	616	Ref.		Ref.	
Employed	60 (46.9)	68 (53.1)	128	1.55 (1.06–2.27)	0.024	1.54 (0.95–2.50)	0.081
**Household income**							
50,000–75,000	182 (59.1)	126 (40.9)	308	Ref.		Ref.	
≥ 75,000	234 (53.7)	202 (46.3)	436	1.25 (0.92–1.67)	0.143	1.22 (0.84–1.78)	0.294

## Discussion

Globally, malnutrition is estimated to be the underlying cause of 45% of all deaths in children under the age of 5 years [18]. The first 2 years of life are crucial as during this period, the body lays the foundation for future growth and development of a child. Any nutritional deficiencies during this time can be manifested in the form of impaired cognitive development, compromised educational achievement and ultimately low economic productivity later in life [19]. The Lancet Maternal and Child Undernutrition series, along with a growing body of evidence, has identified the need to focus on the first 1000 days window of opportunity and the consumption of a diverse diet, including an adequate number of food items and food groups to prevent malnutrition among children [18, 20–25]. Irrespective of this guidance, the consumption of a diverse diet (44.1%) and an adequate number of food groups (84.7%) was found to be low in our study population. This was similar to other developing countries in the region, such as Afghanistan (MDD 22%, MMF 51%), Bangladesh (MDD 27%, MMF 64%), India (MDD 20%, MMF 36%) and Nepal (MDD 45%, MMF 71%) [26–32]. Complementary foods primarily consumed by infants and young children were found to be grains, tubers and roots with limited intake of flesh foods and fruits and vegetables – resembling the dietary patterns in other South Asian countries [33]. A meta-analysis of IYCF practices and child growth outcomes from eight countries in South Asia found that poor diets among children 6–23 months were a primary factor of child stunting [33]. Additionally, children whose diets did not meet the MDD were more likely to be wasted, while children who did not meet MMF were more likely to be severely wasted [34]. Hence, affirming the urgency of public health professionals to emphasis the need to improve complementary feeding practices in Pakistan [35, 36].

Our study found a gradual increase in the likelihood of children meeting the MDD at 9 months, 12 months, 18 months, and 24 months as compared to children at 6 months (reference group). Children aged 9–24 months were twenty times more likely to meet MDD as compared to their younger selves at 6 months of age. A similar association between dietary diversity and the child’s age were observed by Aguayo et al. in four studies exploring the determinants of IYCF practices from countries within the region [27, 36–38]. Furthermore, these studies identified that IYCF practices were more likely to be suboptimal among first-born children, children with younger mothers, children whose mothers were less educated and unemployed and children living in poorer households. These findings are similar to our univariate analysis, which identified that mothers with higher levels of education and employment were more likely to feed their children a diversified diet. However, unlike Aguayo et al., our multivariable analysis found no significant association between maternal education and employment status on MDD in children 6–23 months. Other factors such as maternal age, birth order, gender and household income also had no significant association on dietary diversity in our study population. Recent studies have found that higher total household expenditures and wealth quintile are a prerequisite of a household’s ability to acquire nutritious foods and achieve dietary diversity [39, 40]. This may indicate that affordability of nutritious and diverse foods may not have been a barrier for our study population.

### Strengths and limitations

A key strength of our study is the low rate of loss to follow-up, which improves the internal validity of the study estimates. Our analysis is limited by the longitudinal cohort nature of our data, which does not permit the assessment of causality. The 24-h recall method used to collect dietary diversity data was a challenging task as some mothers may have had difficulty in recollected the exact details of food consumption due to forgetfulness. Subjects with any missing data were excluded from all models. Despite these limitations, our study identified determinants of IYCF practices among urban mothers.

## Conclusion

Our study revealed that Infant and young child feeding practices are significantly associated with maternal education employment, and the child’s age. Therefore, it is essential that investments be made towards protective breastfeeding and complementary feeding policies and legislations, female education and ensuring the availability of affordable nutritious and diverse foods.

## Data Availability

The datasets used for the article and the study is available from the corresponding author on request.

## Abbreviations

MBRCS: Multi-Country Body Composition Reference Study
IYCF: Infant and young child nutrition
DDS: Dietary diversity score
IAEA: International Atomic Energy Agency
MDD: Minimum dietary diversity
MAD: Minimum acceptable diet
MMF: Minimum meal frequency
WHO: World Health Organization

## References

[1] United Nations Children’s Fund, World Health Organization, The World Bank (2020). UNICEF-WHO-World Bank joint child malnutrition estimates.

[2] Government of Afghanistan (2019). Afghanistan Health Survey (AHS) 2018.

[3] National Institute of Population Research and Training (NIPORT), and ICF (2019). Bangladesh demographic and health survey 2017-18: key indicators.

[4] National Statistics Bureau (2011). Bhutan multiple Indicator survey 2010.

[5] Ministry of Health and Family Welfare (MoHFW), Government of India, UNICEF and Population Council (2019). Comprehensive National Nutrition Survey (CNNS) National Report.

[6] Ministry of Health, Nepal; New ERA; and ICF (2017). Nepal demographic and health survey 2016.

[7] Government of Pakistan (2019). National Nutrition Survey 2018–19.

[8] Department of Census and Statistics (DCS) and Ministry of Health, Nutrition and Indigenous Medicine (2017). Sri Lanka demographic and health survey 2016. DCS and ministry of health.

[9] Torlesse H, Aguayo VM (2018). Aiming higher for maternal and child nutrition in South Asia. Matern Child Nutr.

[10] Pan American Health Organization and World Health Organization (2003). Guiding principles for complementary feeding of the breastfed child.

[11] Black RE, Allen LH, Bhutta ZA (2008). Maternal and child undernutrition: global and regional exposures and health consequences. Lancet.

[12] Government of Pakistan (2018). Complementary feeding practices in Pakistan: an in-depth analysis of PDHS 2012–13.

[13] WHO (2010). Indicators for assessing infant and young child feeding practices - part 2 measurement.

[14] Woon FC, Chin YS, Ismail IH, Chan YM, Batterham M, Latiff AHA (2018). Contribution of early nutrition on the development of malnutrition and allergic diseases in the first year of life: a study protocol for the mother and infant cohort study (MICOS). BMC Pediatrics.

[15] Doctoral CRP on Longitudinal Measures of Body Composition of Healthy Infants and Young Children up to 2 Years of Age Using Stable Isotope Techniques https://www.iaea.org/events/evt1805209.

[16] Butte NF (2000). Body composition during the first 2 years of life: an updated reference. Pediatr Res.

[17] Gibson RS, Ferguson EL (2008). An interactive 24-hour recall for assessing the adequacy of iron and zinc intakes in developing countries.

[18] Black RE, Victora CG, Walker SP, Bhutta ZA, Christian P, De Onis M (2013). Maternal and child undernutrition and overweight in low-income and middle-income countries. Lancet.

[19] Prado EL, Dewey KG (2012). Nutrition and brain development in early life. Nutr Rev.

[20] Bhutta ZA, Ahmed T, Black RE, Cousens S, Dewey K, Giugliani E (2008). What works? Interventions for maternal and child undernutrition and survival. Lancet.

[21] Bryce J, Coitinho D, Darnton-Hill I, Pelletier D, Pinstrup-Andersen P, Maternal and Child Undernutrition Study Group (2008). Maternal and child undernutrition: effective action at national level. Lancet.

[22] Morris SS, Cogill B, Uauy R, Maternal Group CUS (2008). Effective international action against undernutrition: why has it proven so difficult and what can be done to accelerate progress?. Lancet.

[23] Victora CG, Adair L, Fall C, Hallal PC, Martorell R, Richter L (2008). Maternal and child undernutrition: consequences for adult health and human capital. Lancet.

[24] Alderman H, Elder L, Goyal A, Herforth A, Hoberg YT, Marini A (2013). Improving nutrition through multisectoral approaches.

[25] Becquey E, Martin-Prevel Y, Traissac P, Dembélé B, Bambara A, Delpeuch F (2010). The household food insecurity access scale and an index-member dietary diversity score contribute valid and complementary information on household food insecurity in an urban West-African setting, 2. J Nutr.

[26] Dizon F, Herforth A, Wang Z (2019). The cost of a nutritious diet in Afghanistan, Bangladesh, Pakistan, and Sri Lanka. Global Food Security.

[27] Na M, Aguayo VM, Arimond M, Mustaphi P, Stewart CP (2018). Predictors of complementary feeding practices in Afghanistan: analysis of the 2015 demographic and health survey. Matern Child Nutr.

[28] Campbell RK, Aguayo VM, Kang Y, Dzed L, Joshi V, Waid J, Guupta SD, Haselow N, West KP (2018). Infant and young child feeding practices and nutritional status in Bhutan. Matern Child Nutr.

[29] Senarath U, Godakandage SSP, Jayawickrama H, Siriwardena I, Dibley MJ (2012). Determinants of inappropriate complementary feeding practices in young children in Sri Lanka: secondary data analysis of demographic and health survey 2006–2007. Matern Child Nutr.

[30] Rah JH, Akhter N, Semba RD, de Pee S, Bloem MW, Campbell AA, Moench-Pfanner R, Sun K, Badham J, Kraemer K (2010). Low dietary diversity is a predictor of child stunting in rural Bangladesh. Eur J Clin Nutr.

[31] Khanal V, Sauer K, Zhao Y (2013). Determinants of complementary feeding practices among Nepalese children aged 6–23 months: findings from demographic and health survey 2011. BMC Pediatr.

[32] Dhami MV, Ogbo FA, Osuagwu UL, Agho KE (2019). Prevalence and factors associated with complementary feeding practices among children aged 6–23 months in India: a regional analysis. BMC Public Health.

[33] Aguayo VM (2017). Complementary feeding practices for infants and young children in South Asia. A review of evidence for action post-2015. Matern Child Nutr.

[34] Harding KL, Aguayo VM, Webb P (2018). Birthweight and feeding practices are associated with child growth outcomes in South Asia. Matern Child Nutr.

[35] Menon P (2012). The crisis of poor complementary feeding in South Asia: where next?. Matern Child Nutr.

[36] Na M, Aguayo VM, Arimond M, Stewart CP (2017). Risk factors of poor complementary feeding practices in Pakistani children aged 6–23 months: a multilevel analysis of the demographic and health survey 2012–2013. Mater Child Nutr.

[37] Na M, Aguayo VM, Arimond M, Narayan A, Stewart CP (2018). Stagnating trends in complementary feeding practices in Bangladesh: an analysis of national surveys from 2004-2014. Matern Child Nutr.

[38] Na M, Aguayo VM, Arimond M, Dahal P, Lamichhane B, Pokharel R (2018). Trends and predictors of appropriate complementary feeding practices in Nepal: an analysis of national household survey data collected between 2001 and 2004. Matern Child Nutr.

[39] Thorne-Lyman AL, Valpiani N, Sun K, Semba RD, Klotz CL, Kraemer K (2010). Household dietary diversity and food expenditures are closely linked in rural Bangladesh, increasing the risk of malnutrition due to the financial crisis. J Nutr.

[40] Torheim LE, Ouattara F, Diarra MM, Thiam FD, Barikmo I, Hatloy A (2004). Nutrient adequacy and dietary diversity in rural Mali: association and determinants. Eur J Clin Nutr.

